# Amygdala nuclei are not uniformly vulnerable to FTLD‐tauopathies in PPA

**DOI:** 10.1002/alz70855_107447

**Published:** 2025-12-25

**Authors:** Rachel M Keszycki, Ivan Ayala, Antonia Zouridakis, Allegra Kawles, Alyssa Macomber, Caroline M Nelson, Grace Minogue, Sandra Weintraub, Rudolph J Castellani, Marsel Mesulam, Changiz Geula, Tamar Gefen

**Affiliations:** ^1^ Northwestern University Feinberg School of Medicine, Chicago, IL, USA; ^2^ Brown University, Providence, RI, USA; ^3^ Mesulam Center for Cognitive Neurology & Alzheimer's Disease, Chicago, IL, USA

## Abstract

**Background:**

Primary progressive aphasia (PPA) is a language‐based dementia often caused by frontotemporal lobar degeneration with tau pathology (FTLD‐tau) of the 3R or 4R species. The amygdala, a limbic region comprised of several nuclei, is implicated in and differentially vulnerable to neurodegenerative diseases. This study investigated whether amygdala nuclei show distinct patterns of pathogenesis in PPA due to underlying 3R vs. 4R FTLD‐tau.

**Method:**

PPA cases with autopsy‐confirmed 3R (*N* = 4) or 4R (*N* = 3) FTLD‐tau and controls (*N* = 3) were identified from the Northwestern Alzheimer's Disease Research Center Brain Bank. Coronal 40‐μm amygdala sections were cut from left hemisphere (*N* = 3 controls, *N* = 2 PPA/3R), right hemisphere (*N* = 2 PPA/3R), or bilaterally (*N* = 3 PPA/4R) based on tissue availability. Sections were stained with Cresyl violet for neurons (all cases) or AT‐8 for phosphorylated tau (PPA only). We quantified neurons and tau inclusions stereologically (StereoInvestigator, MicroBrightField) and %AT‐8 immunopositivity digitally (HALO, Indica Labs) in the lateral, basal, accessory basal, and central nuclei. Multiple linear regressions covaried for hemisphere and clinical (PPA, controls) or pathological (3R, 4R, controls) diagnosis with pathological marker as the outcome.

**Result:**

The basal nucleus showed significant neuronal loss in PPA versus controls (*p* <0.05). It also had the most total tau inclusions, significantly more than the accessory basal (*p* <0.01–0.05) and central (both *p* <0.05) nuclei. The lateral nucleus of PPA/3R cases demonstrated significant neuronal loss versus other nuclei (*p* <0.01–0.05) and pathologic groups (both *p* <0.01). PPA/3R showed higher total tau inclusions than PPA/4R across all nuclei (*p* <0.01–0.05) due to prevalent intraneuronal inclusions. In contrast, PPA/4R had more glial pathology, particularly in the central nucleus (*p* <0.05). Left %AT‐8 was greater than right in bilaterally sectioned PPA/4R cases (*p* <0.001).

**Conclusion:**

Findings highlight distinct vulnerabilities of amygdala nuclei in PPA due to FTLD‐tauopathies. Irrespective of tauopathy, the basal nucleus showed the greatest neuronal loss and tau pathology in PPA. PPA/3R showed more global tau accumulation and lateral nucleus neuronal loss, whereas PPA/4R demonstrated greater central nucleus glial tau accumulation. Results also demonstrate leftward asymmetry of pathogenesis in PPA, even in limbic regions. Future investigation of neuroinflammation and synaptic markers may yield additional insights into these vulnerabilities.

